# Predicting Phenotype and Emerging Strains among *Chlamydia trachomatis* Infections

**DOI:** 10.3201/eid1509.090272

**Published:** 2009-09

**Authors:** Deborah Dean, William J. Bruno, Raymond Wan, João P. Gomes, Stéphanie Devignot, Tigist Mehari, Henry J.C. de Vries, Servaas A. Morré, Garry Myers, Timothy D. Read, Brian G. Spratt

**Affiliations:** Children’s Hospital Oakland Research Institute, Oakland, California, USA (D. Dean, R. Wan, T. Mehari); University of California, San Francisco, California, USA (D. Dean); University of California, Berkeley, California, USA (D. Dean); Los Alamos National Laboratories, Los Alamos, New Mexico, USA (W.J. Bruno); National Institute of Health, Lisbon, Portugal (J.P. Gomes); Institut de Médecine Tropicale du Service de Santé des Armées, Marseille, France (S. Devignot); University of Amsterdam, the Netherlands (H.J.C. de Vries); Vrije Universiteit Medical Center, Amsterdam (S.A. Morré); University of Maryland School of Medicine, Baltimore, Maryland, USA (G. Myers); Emory University, Atlanta, Georgia, USA (T.D. Read); Imperial College, London, UK (B.G. Spratt)

**Keywords:** Chlamydia trachomatis, single nucleotide polymorphisms, bacterial sequence typing, sexually transmitted infections, trachoma, bacteria, research

## Abstract

*Chlamydia trachomatis* is a global cause of blinding trachoma and sexually transmitted infections (STIs). We used comparative genomics of the family *Chlamydiaceae* to select conserved housekeeping genes for *C. trachomatis* multilocus sequencing, characterizing 19 reference and 68 clinical isolates from 6 continental/subcontinental regions. There were 44 sequence types (ST). Identical STs for STI isolates were recovered from different regions, whereas STs for trachoma isolates were restricted by continent. Twenty-nine of 52 alleles had nonuniform distributions of frequencies across regions (p<0.001). Phylogenetic analysis showed 3 disease clusters: invasive lymphogranuloma venereum strains, globally prevalent noninvasive STI strains (*ompA* genotypes D/Da, E, and F), and nonprevalent STI strains with a trachoma subcluster. Recombinant strains were observed among STI clusters. Single nucleotide polymorphisms (SNPs) were predictive of disease specificity. Multilocus and SNP typing can now be used to detect diverse and emerging *C. trachomatis* strains for epidemiologic and evolutionary studies of trachoma and STI populations worldwide.

## Abstract 2

Single nucleotide polymorphisms can be used for epidemiologic and evolutionary studies worldwide.

*Chlamydia trachomatis* is spread by close social contact or sexual activity. Worldwide, *C. trachomatis* is the leading preventable cause of blindness and bacterial sexually transmitted infections (STIs). Various typing techniques have been developed to better understand the epidemiology and pathogenesis of chlamydial diseases. Early typing schemes used monoclonal and polyclonal antibodies directed against the major outer membrane (MOMP) (*1*), and differentiated the organism into serovars and seroclasses: B class (comprising serovars B, Ba, D, Da, E, L_2_, L_2_a), C class (A, C, H, I, Ia, J, Ja, K, L_1_, L_3_), and intermediate class (F, G). Sequencing of *ompA*, which encodes MOMP, has refined typing, detecting numerous trachoma (*2*,*3*) and sexually transmitted (*4*,*5*) serovar subtypes.

Seroclasses, however, do not correlate with disease phenotypes. For example, A, B, Ba, and C are responsible for trachoma, whereas lymphogranuloma venereum (LGV) strains, L_1-3_, are associated with invasive diseases, such as suppurative lymphadenitis and hemorrhagic proctitis (*6*). Other typing techniques, such as restriction fragment length polymorphism ( (*7*), random amplification of polymorphic DNA, or pulsed-field gel electrophoresis (PFGE) (*8*), and amplified fragment length polymorphism (AFLP) (*9*) also correlate poorly with disease phenotype, and none have been standardized across laboratories.

Multilocus sequence typing (MLST) has been used to characterize strains and lineages of numerous pathogens associated with human diseases that cause serious illness and death, including *Neisseria meningitidis*, *Staphylococcus aureus*, *Vibrio cholerae*, and *Haemophilus influenzae*. MLST uses 500–700 bp sequences of internal regions of 6–8 housekeeping genes, excluding genes suspected to be under immune selection (where there is positive selection for sequence diversity) and ribosomal RNA genes (which are multicopy and too conserved) (*10*). Advantages of MLST include its precision, allowing simple interlaboratory comparisons, good discrimination between strains, and buffering against the distorting effect of recombination on genetic relatedness. MLST data are also amenable to various population genetic analyses (*11*,*12*). Databases for >30 species are curated at www.mlst.net and pubmlst.org. In parallel with our study, 2 multilocus schemes have recently been developed for *C. trachomatis*. The first violated the above premise by including *ompA*, which is under immune selection (*13*). The second included only laboratory-adapted and 5 clinical E strains from the Netherlands (*14*).

In this work, unlike the other *C. trachomatis* MLST schemes, we used complete genomic comparisons of 7 strains from 4 species within the family *Chlamydiaceae* to identify conserved candidate housekeeping genes across the genomes. This approach ensures that the chosen loci are stable over the course of evolution, and allows for future application of a unified MLST scheme for other *Chlamydiaceae* spp. We typed a diverse worldwide collection of reference and clinical isolates from trachoma and STI populations, correlating genetic variation with geography and disease phenotype. We found disease-specific single nucleotide polymorphisms (SNPs) and a diversity of new strains including recombinant strains that occurred for *ompA* relative to housekeeping loci, following up on our recent discovery of this phenomenon at multiple loci in *Chlamydiaceae* genomes (*15*–*17*).

## Methods

### Reference and Clinical Samples

Nineteen *C. trachomatis* reference strains (A/SA-1, B/TW-5, Ba/Apache-2, C/TW-3, D/UW-3, Da/TW-448, E/Bour, F/IC-Cal3, G/UW-57, H/UW-4, I/UW-12, Ia/IU-4168, J/UW-36, Ja/UW-92, K/UW-31, L1/440, L2/434, L2a/TW-396, and L3/404) and 68 clinical isolates from 6 geographic locations worldwide (obtained from patients with trachoma and STIs including proctitis) were analyzed. Because de-identified clinical data and samples were used, the research was considered institutional review board exempt by Children’s Hospital Oakland Research Institute.

### Selection of Housekeeping Genes

**Figure 1:**
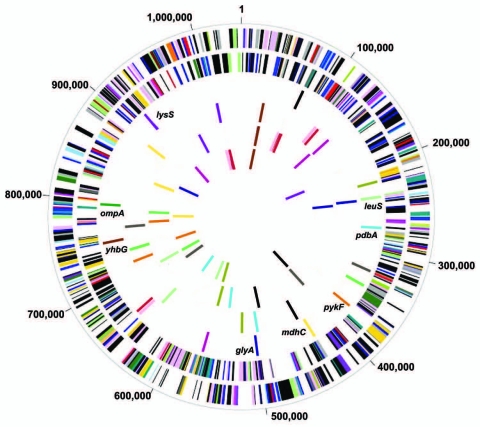
Comparison of 14 housekeeping genes among genome sequences of 4 *Chlamydiaceae* species and 7 strains. Circle 1, genes on forward *Chlamydia trachomatis* strand, color coded by role category; Circle 2, genes on reverse *C. trachomatis* strand; Circle 3, multilocus sequence typing (MLST) candidates, *C. trachomatis*; Circle 4, MLST candidates, *C. pneumoniae* AR39; Circle 5, MLST candidates, *C. caviae* (GPIC); Circle 6, MLST candidates, *C. muridarum* (MoPn). Colors in circles 3, 4, 5 and 6 are consistent for each gene across genomes i.e., “blue” gene in each circle is ortholog in that genome for “blue” gene in *C. trachomatis*. **Blue, *glyA*, serine hydroxymethyl-transferase**; red, tryptophanyl-tRNA synthetase; **yellow*, mdhC,* malate dehydrogenase**; green, V-type ATPase, subunit A; **cyan, *pdhA,* pyruvate dehydrogenase**; black, GTP-binding protein lepa; magenta, transcription termination factor rho; **brown, *yhbG*, probable ABC transporter ATP-binding protein**; **orange, *pykF*, pyruvate kinase**; olive green, conserved hypothetical protein; gray, acetyl-CoA carboxylase beta subunit; pink, threonyl-tRNA synthetase; **violet, *lysS*, lysyl-tRNA synthetase**; **light green*, leuS,* leucyl-tRNA synthetase**. Those denoted in **boldface** above were used for *C. trachomatis* MLST. *ompA* gene location is shown for *C. trachomatis* (dark green).

We genome-sequenced 7 strains from 4 species of the 2 genera of *Chlamydiaceae*: *C. trachomatis* (strains D/UW-3/CX [*18*] and A/Har-13 [*19*]), *Chlamydia muridarum* (rodent strain MoPn [*20*]), *Chlamydophila pneumoniae* (human strains AR39 [*20*]; CWL029 [*21*], and J138 [*21*]), and *Chlamydophila caviae* (guinea pig inclusion conjunctivitis strain [*22*]), the most distantly related species of *Chlamydiaceae*. On the basis of comparative genomics (*20*) and comparisons generated by CGView (*23*), we identified an initial candidate pool of 14 housekeeping genes (Figure 1) present in all 7 genomes with an average BLAST score ratio (BSR) (*24*) >0.5 for orthologs queried against *C. caviae* relative to the BLAST score of each sequence against itself. The BSR of >0.5 provides a cutoff to select genes that have lower levels of nucleotide sequence divergence in the genome (i.e., putative housekeeping genes). We then selected 7 genes (Figure 1) on the basis of i) diverse chromosomal regions where a single recombinational exchange would be unlikely to co-introduce >1 selected gene; ii) regions where several contiguous genes were involved in metabolic or key functions; iii) essential metabolic enzymes (e.g., tRNA synthases); iv) genes without similarity to human genes; and v) no genes under diversifying selection.

### *ompA* and MLST Analyses

DNA was extracted from isolates using Roche High Pure Kits (Roche Diagnostics, Pleasanton, CA, USA) and *ompA* genotyped as described (*15*,*16*,*25*). DNASTAR (Madison, WI, USA) was used to design primers to amplify ≈600–700 bp for each gene (Table 1**)**; BLAST (NCBI; http://blast.ncbi.nlm.nih.gov/Blast.cgi) was used to ensure primer specificity for *C. trachomatis* genes. MLST PCR was carried out in 96-well plates as described (*26*). Sequenced DNA (GenBank accession nos. FJ45414–FJ746022) using ABI3700 instruments were aligned by using MegAlign (DNASTAR). Each unique sequence for a locus was designated as a unique allele using Sequence Output (www.mlst.net). Each allelic profile (made up of the string of integers corresponding to allele numbers at the 7 loci) was assigned as a different strain or clone and given an ST as a clone descriptor. All STs have been deposited in the *C. trachomatis* site at www.mlst.net.

**Table 1:** Primer pairs used for PCR of chlamydiaceae species and strains

Locus	Region	Primer name	Sequence (5′ → 3′)	Length of sequence, bp
*glyA*	CT432	FglyA	GAAGACTGTGGCGCTGTTTTATGG	522
		RglyA	CTTCCTGAGCGATCCCTTCTGAC	
*mdhC*	CT376	FmdhC	GGAGATGTTTTTGGCCTTGATTGT	519
		RmdhC	CGATTACTGCACTACCACGACTCT	
*pdhA*	CT245	FpdhA	CTACAGAAGCCCGAGTTTTT	549
		RpdhA	CTGTTTGTTGCATGTGGTGATAAG	
*yhbG*	CT653	FyhbG	TCAAGTCAATGCAGGAGAAAT	504
		RyhbG	GATAGTGTTGACGTACCATAGGAT	
*pykF*	CT332	FpykF	ATCTTATCGCTGCTTCGTT	525
		RpykF	cagcaataatagggagata	
*lysS*	CT781	FlysS	GAAGGAATCGATAGAACGCATAAT	576
		RlysS	ATACGCCGCATAACAGGGAAAAAC	
*leuS*	CT209	FleuS	TCCCTTGGTCGATCTCCTCAC	519
		RleuS	GGGCATCGCAAAAACGTAAATAGT	

Allelic profiles and concatenated sequences were used to determine the relatedness of isolates. Average pairwise diversity between isolates was calculated from the 3,714-bp concatenated sequence of the 7 loci for each isolate joined in-frame using MEGA4 (*27*). Synonymous (dS) and nonsynonymous (dN) substitutions were determined using MEGA4 for each locus. Allele frequencies per locus and geographic region were calculated using SAS software 9.2 (SAS Institute, Inc., Cary, NC, USA) with the PROC FREQ tool supplying the frequency count. We calculated a classification index (*11*) on the basis of allele and ST frequency between populations of different geographic regions to determine the probability of association of an allele with a particular continental/subcontinent region. Statistical significance was determined by 10,000 resamplings of allele and ST frequency per region.

### Strain Clustering and Single Nucleotide Polymorphism Analyses

eBURST (www.mlst.net) was used to identify clusters of related and singleton STs that were not closely related to any other ST (*12*) and to predict patterns of evolutionary descent. MEGA4 (*27* )was used to construct a tree from concatenated sequences by using minimum evolution, neighbor joining, or unweighted pair group method with arithmetic mean, with various substitution models including Kimura 2-parameter, Jukes Cantor, and p-distance; 1,000 bootstrap replicates were used to test support for each node in the tree. The short evolutionary distances (<≈0.01) imply that back-substitutions were rare, and as expected, all methods gave similar results (data not shown). SplitsTree (www.splitstree.org) was used for evolutionary tree construction by decomposition analyses using the distance matrix produced from pairwise comparisons of concatenated sequences to determine interconnected networks (*28*).

A matrix of all SNPs by ST was produced in Excel. SAS was used to identify which SNPs were associated with an ST using PROC FREQ. Statistical significance was determined by using a classification index as above for the probability of association of a SNP with a particular ST. Levene’s test (*29*) was used to determine whether there was equal variance across the 87 isolates. A p value of <0.05 was considered significant.

## Results

### Discrimination of *C. trachomatis* by MLST

Figure 1 shows genomic alignments for *C. trachomatis* (D/UW-3/CX), *C. muridarum*, *C. pneumoniae* (AR39), and *C. caviae*. The *C. trachomatis* (A/Har-13 and D/UW-3/CX) and *C. pneumoniae* (AR39, CWL029, and J138) genome sequences were almost identical within species for gene content and could be represented by D/UW-3/CX and AR39, respectively.

*ompA* genotypes were compared with STs resolved by MLST. The Technical Appendix shows *ompA* genotype (first letter of strain ID), ST, assigned alleles, and clinical characteristics for each isolate. There were 44 STs (0.51 ST/isolate) for the 87 isolates. Thirty STs were represented by a single isolate. In some cases, STI isolates from diverse geographic regions shared the same ST. For example, *ompA* genotype E STI isolates (ST39) were found in California, USA; Amsterdam, the Netherlands; Ecuador; Lisbon, Portugal; and Tanzania. Similarly, LGV genotypes (ST1) were identified in San Francisco, California, USA; Seattle, Washington, USA; and Amsterdam; 2 clinical L_2_b genotypes (ST33) were restricted to Amsterdam. In contrast, no trachoma isolates from different continents shared the same ST.

*ompA* genotypes correlated poorly with relatedness between strains by MLST data (Technical Appendix). Isolates of the same ST had up to 4 different *ompA* genotypes. For example, ST19 included *ompA* genotypes D, H, I, and J. For each *ompA* genotype, 38%–100% belonged to different STs. Different STs with the same *ompA* genotype were closely related by MLST (e.g., isolates with C and F *ompA* genotypes); others were not. Isolates of D, E, and Ja *ompA* genotypes differed at as many as 5 MLST loci.

### Allele Characteristics and Localization by Geography

Allele characteristics are shown in Table 2. The number of alleles at each locus varied from 4 to 11. The average pairwise distance and dS and dN are provided. We determined allele frequencies on the basis of continental/subcontinental regions (Table 3). The majority of alleles were observed multiple times. Seventeen were found only once, and 28 were unique to a specific region (Table 3). The range was from 1 allele at the *lysS* locus for South America to 9 in North America. The highest frequency of a unique allele was 84.6% (*leuS* allele 7) for Asia, which also had the highest proportion of unique alleles, 6/17 (35.29%). There was a significant nonuniform distribution of alleles at each locus by classification index.

**Table 2:** Characteristics of alleles for each locus

Gene locus	No. alleles	Length, bp	No. polymorphic sites	Average pairwise distance	Average dS	Average dN
*glyA*	7	522	5	0.003	0.0101	0.0034
*mdhC*	4	519	3	0.001	0.0112	0.0025
*pdhA*	7	549	6	0.0003	0.0076	0.0030
*yhbG*	8	504	21	0.01	0.0670	0.0026
*pykF*	7	525	7	0.003	0.0105	0.0034
*lysS*	8	576	9	0.002	0.0093	0.0044
*leuS*	11	519	10	0.003	0.0104	0.0055
Overall	52	3,714	61	0.003		

**Table 3:** Allele frequencies by geographic region by locus

Gene locus	No. alleles	Allele frequency, no. (%)*						Classification index p value
		Africa (n = 11)	Northern Europe (n = 14)	Southern Europe (n = 10)	Asia (n = 13)	North America (n = 33)	South America (n = 6)	
*glyA*	7	3 (90.9) 6 (9.1)	1 (7.1) 3 (42.9) **4 (7.1)** **5 (14.3)** 6 (28.6)	3 (60) 6 (40)	3 (30.8) 6 (7.7) **7 (61.5)**	1 (15.1) **2 (3.0)** 3 (54.6) 6 (27.3)	3 (33.3) 6 (66.7)	<0.001
*mdhC*	4	3 (90.9) 4 (9.1)	1 (7.1) **2 (14.3)** 3 (64.3) 4 (14.3)	3 (80) 4 (20)	3 (100)	1 (18.2) 3 (72.7) 4 (9.1)	3 (50.0) 4 (50.0)	<0.001
*pdhA*	7	**1 (9.1)** 3 (90.9)	**2 (7.1)** 3 (92.9)	3 (60) **4 (30)** **7 (10)**	3 (100)	3 (94.0) **5 (3.0)** 6 (3.0)	3 (100.0)	<0.001
*yhbG*	8	2 (9.1) 6 (90.9)	2 (28.6) 3 (7.1) 6 (42.7) 8 (21.4)	2 (40) 6 (50) **7 (10)**	**1 (7.7)** **4 (7.7)** 5 (7.7) 6 (76.9)	2 (21.2) 3 (3.0) 5 (3.0) 6 (57.6) 8 (15.2)	2 (66.7) 6 (33.3)	<0.001
*pykF*	7	3 (81.8) 6 (9.1) 7 (8.1)	1 (12.5) 6 (50) 7 (37.5)	6 (60) 7 (40)	3 (92.3) 7 (7.7)	1 (18.2) **2 (9.1)** **4 (3.0)** **5 (3.0)** 6 (39.4) 7 (27.3)	6 (33.3) 7 (66.7)	<0.001
*lysS*	8	4 (15.2) 5 (72.7) **7 (9.1)**	1 (14.3) 4 (78.6) 8 (7.1)	4 (70) 8 (30)	4 (7.7) 5 (30.8) **6 (61.5)**	1 (3.0) **3 (3.0)** 4 (75.8) 8 (18.2)	**2 (16.7)** 4 (66.7) 8 (16.7)	<0.001
*leuS*	11	2 (9,1) 3 (9.1) 9 (81.8)	3 (57.1) **6 (21.4)** 11(21.4)	3 (80) **4 (10)** **5 (10)**	2 (7.7) **7 (84.6)** **10 (7.7)**	**1 (3.0)** 3 (48.5) **8 (30.3)** 9 (3.0) 11 (15.2)	3 (100.0)	<0.001
Total no. alleles	52	17	24	17	17	31	13	

*Numbers are arranged vertically for each locus to represent the individual alleles (e.g., glyA, alleles are assigned 1, 2, 3, 4, 5, 6 and 7 because there are 7 alleles for this locus). Alleles marked in **boldface** are specific for a single geographic region. n values indicate number of samples.

### Phylogenetic Grouping of STs by Disease Phenotypes and Evidence for Recombination

**Figure 2:**
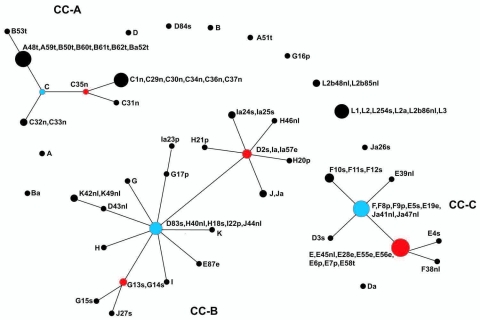
eBURST population snapshot for *Chlamydia trachomatis* isolates. Sequence types (STs) that are linked differ at single multilocus sequence typing locus and represent clonal complexes. Strains in the same clonal complexes are likely descended from the same recent ancestor. Each circle represents a ST. An ST in blue is most likely the primary founder of the clonal complex; STs in red are subgroup founders. The number of isolates of each ST is represented by the area of the circle. Clonal complex A (CC-A) for trachoma strains; CC-B, noninvasive, nonprevalent urogenital strains; and CC-C, noninvasive, globally prevalent urogenital strains. The eBURST report is shown in the Technical Appendix.

eBURST (*11*,*12*) generated 3 clonal complexes (CC) (Figure 2): trachoma strains, A, B, Ba, and C (CC-A); noninvasive STIs with low population prevalence (CC-B); and noninvasive, globally prevalent D/Da, E, and F STIs (CC-C). The Technical Appendix shows eBURST data, including single, double, and triple locus variants (S/D/TLVs).

**Figure 3:**
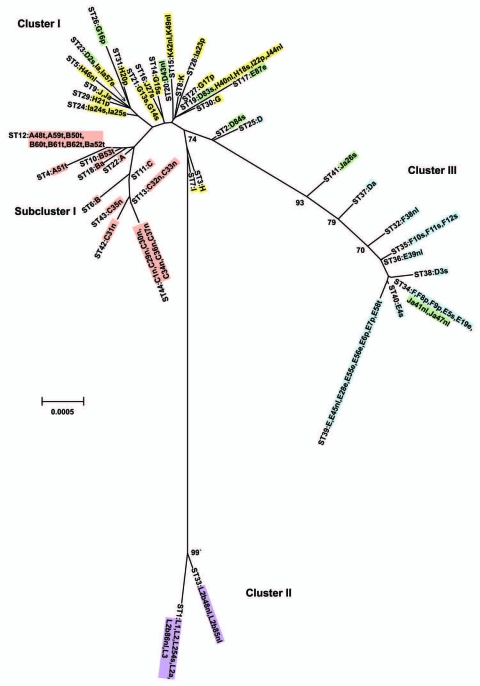
Minimum evolution tree. The tree was constructed using the matrix of pairwise differences between the 87 concatenated sequences for the 7 loci using maximum composite likelihood method for estimating genetic distances. Numbers are bootstrap values (1,000 replicates) >70%. Lavender, invasive lymphogranuloma venereum (LGV); gold, noninvasive, nonprevalent sexually transmitted infection (STI) strains; red, trachoma strains; blue, noninvasive, highly prevalent STI strains; green, putative recombinant stains. Scale bar indicates number of substitutions per site.

Relationships between the isolates was further explored by constructing a minimum-evolution tree using MEGA4 (*27*). These data showed 3 disease clusters (Figure 3). Cluster I comprised noninvasive STIs (eBURST CC-B) and a trachoma subcluster (eBURST CC-A). Cluster II comprised only invasive LGV strains. Cluster III included noninvasive prevalent D/Da, E and F STIs (eBURST CC-C). E58t strain (ST39; cluster III) was isolated from the conjunctiva of a trachoma patient, most likely representing autoinoculation from the urogenital tract, because all other isolates of this ST were from STIs.

**Figure 4:**
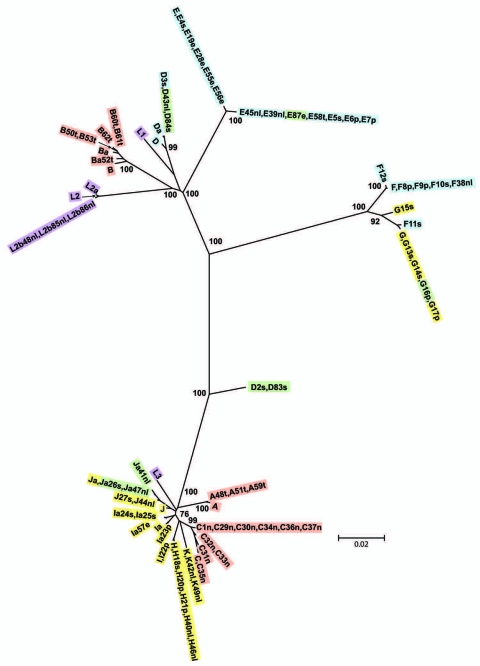
Minimum evolution tree for *ompA*. The tree was constructed using the matrix of pairwise differences between the 87 sequences by using the maximum composite likelihood method for estimating genetic distances. Numbers are bootstrap values (1,000 replicates) >70%. Lavender, invasive lymphogranuloma venereum (LGV); gold, noninvasive, nonprevalent sexually transmitted infection (STI) strains; red, trachoma strains; blue, noninvasive, highly prevalent STI strains; green, putative recombinant stains. Scale bar indicates number of substitutions per site.

Nine isolates did not localize on the MLST tree with strains of the same *ompA* genotype (Figure 3). Ja41nl and Ja47nl, which were expected to cluster with other J and Ja isolates in cluster I if the genome sequences were similar, were identical by MLST to reference strain F and clinical isolates F8p, F9p, E19e, and E5s in cluster III. Similarly, D83s, which were expected to cluster with other D and Da isolates in cluster III, had the same ST as H40nl, H18s, I22p, and J44nl in cluster I; D2s were identical to Ia and Ia57e in cluster I. Additionally, G16p did not cluster with the other G isolates in cluster I. In analyzing locations of incongruence between clinical D and E isolates in cluster I, compared with those in cluster III, the loci that differed were *glyA*, *yhbG,* and *pykF* in which allele assignments were identical, in general, to G, H, I, Ia, J, Ja, and K strains (Technical Appendix) in cluster I. These were the exact same loci that differed for Ja41nl and Ja47nl in cluster III, compared with other J/Ja isolates in cluster I. Ja26s differed at *glyA*, *mdhC*, and *yhbG*, whereas G16p differed at *yhbG, lysS*, and *leuS.* Furthermore, the *ompA* tree (Figure 4) was incongruent with the MLST tree. We interpret all 9 isolates to be recombinants.

**Figure 5:**
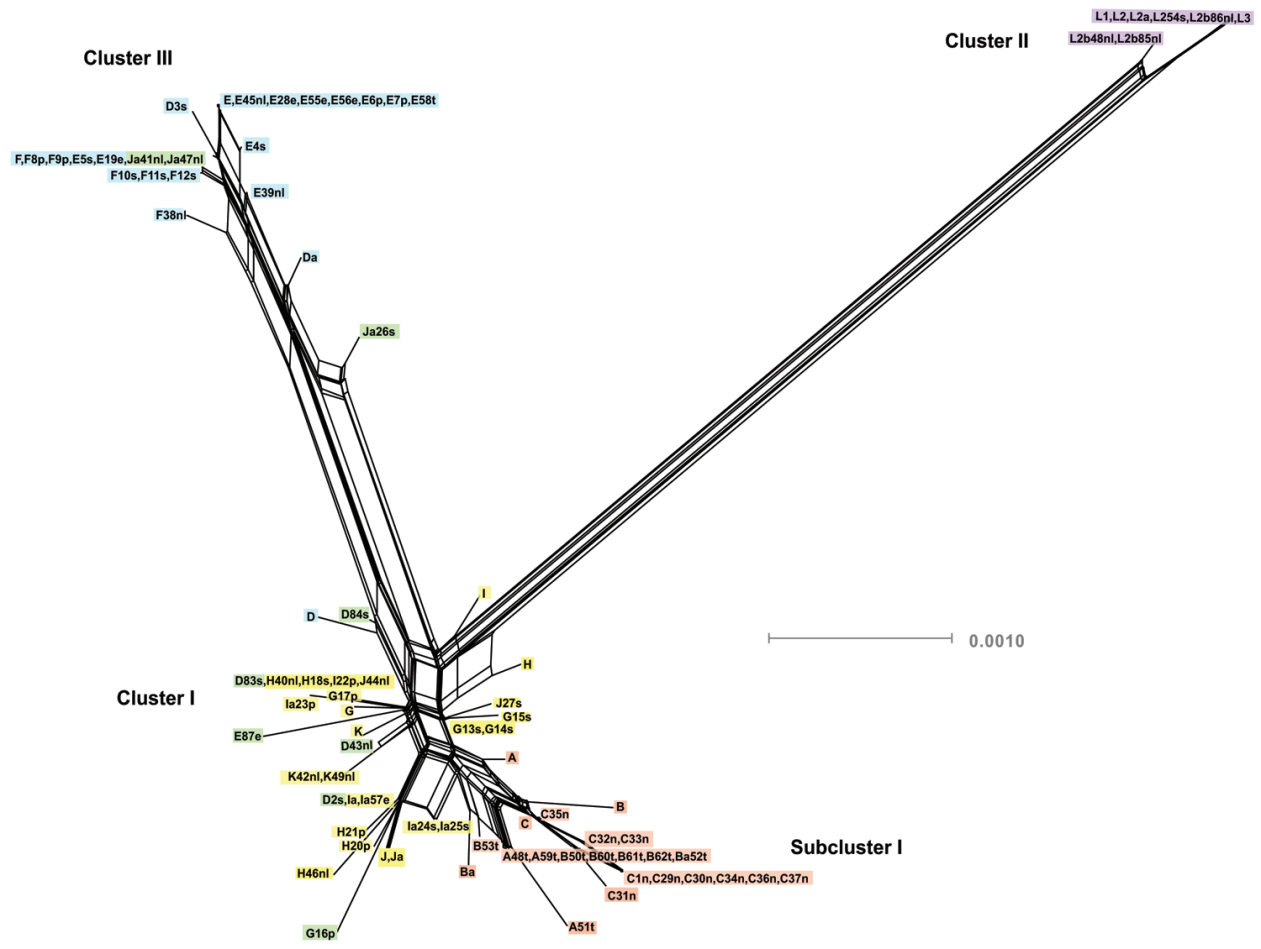
SplitsTree obtained by using concatenated sequences of the 7 loci for the 87 isolates. Cluster I, noninvasive, nonprevalent *Chlamydia trachomatis* strains (gold) with trachoma Subcluster I (red); cluster II, invasive lymphogranuloma venereum (LGV) isolates (purple); and cluster III, noninvasive globally prevalent sexually transmitted infection (STI) strains (blue). Isolates colored green represent putative recombinant strains. Scale bar indicates number of substitutions per site.

SplitsTree decomposition evaluated alternative evolutionary pathways that might indicate recombination between MLST loci (Figure 5). There was considerable network structure, providing evidence of alternative pathways between strains, which may indicate that recombination has influenced the evolution of housekeeping genes for the *C. trachomatis* strains.

### SNPs Associated with Disease Phenotypes

We identified 61 polymorphic sites among the 7 loci. Multiple SNPs were significantly associated with each of the 3 clusters and disease groups (Table 4). For example, 15 SNPs in *yhbG* and *leuS* were 100% specific for all LGV strains in cluster III. Any 1 of these SNPs could be used to identify these strains. SNPs 4, 29, 31, 33, and 34 (together or any 1 alone) were specific for the cluster II STIs. For the trachoma Subcluster I, unlike for other clusters, only SNP 38 was associated with reference strains B and C and all clinical trachoma strains; SNPs 54, 55 and 57 together (but not any alone) represented all trachoma strains except reference strain A. Based on classification indices and Levene’s test, the null hypothesis of a uniform distribution of SNPs was rejected at the respective locus.

**Table 4:** SNPs and combinations of SNPs that correlate 100% with designated cluster and disease phenotype group*

Gene locus	SNP no.	Cluster III	Cluster II	Subcluster I†
*glyA*	1–5		4‡	
*mdhC*	6–8			
*pdhA*	9–14			
*yhbG*	15–35	15§ 20 22–26 30	29‡ 31 33 34	
*pykF*	36–42			38¶
*lysS*	43–51			
*leuS*	52–61	52§ 56 61		54 55 57
Total no. SNPs	61	15	5	4

*SNP, single nucleotide polymorphism. Each SNP is 100% specific for designated cluster and disease group (e.g., SNP 15 identifies cluster III but all 15 SNPs are specific to cluster III). Cluster III comprises all clinical and reference lymphogranuloma venereum (LGV) strains; cluster II comprises all reference and clinical D, Da, E, F, and recombinant clinical Ja strains except recombinant clinical D2s, D43nl, E87e, and reference Ja strains; Subcluster I comprises all reference and clinical trachoma A, B, Ba and C strains except reference strains A and Ba. Disease phenotype groups: invasive LGV strains (cluster III); trachoma A, B, Ba and C strains (Subcluster I); and noninvasive globally prevalent STI D/Da, E, and F strains (cluster II). Clinical Ja strains likley acquired a Ja *ompA* gene by recombination. †SNPs 54, 55, and 57 together (not independently) are specific for all trachoma strains except reference A strain. ‡p<0.01 for classification index. §p<0.001 for classification index. ¶p = 0.008 for classification index.

## Discussion

Accumulating evidence for recombination among *Chlamydiaceae* in general, and *C. trachomatis* in particular, has motivated a typing system that provides buffering from the distorting effects of genetic reshuffling that plague systems based on a single locus. We therefore developed an MLST scheme derived from comparative genomics of species within the family *Chlamydiaceae* to select conserved chromosomally dispersed housekeeping genes. Our scheme showed considerable variability in allelic profiles associated with geographic regions, as well as diverse and recombinant strains. We also identified SNPs that correlated with the 3 *C. trachomatis* disease groups: invasive LGV diseases, noninvasive urogenital diseases, and trachoma.

Comparative genomics of *Chlamydia* and *Chlamydophila* spp. identified 14 conserved housekeeping genes that could be used to extend MLST schemes for these and potentially other *Chlamydiaceae* spp. Surprisingly, each gene was located in a different position within the respective genome, indicating a lack of synteny among chromosomes (*20*) (Figure 1), except for the 2 *C. trachomatis* and 3 *C. pneumoniae* strains, which share within species >99% nucleotide sequence identity. This finding suggests that future schemes should select loci to ensure reasonable coverage of the chromosome.

Although there was relatively little sequence diversity in the housekeeping genes, the number of STs (0.51 ST/isolate) was similar to that of other bacterial pathogens. The previous *C. trachomatis* MLST scheme had 0.60 ST/isolate (*14*). None of the loci were identical to ours. In a recent study of the bacterium *Burkholderia pseudomallei* in Australia, there were 0.65 ST/isolate (*11*,*12*) with relatively little diversity and few alleles per locus. However, high levels of recombination are believed to shuffle alleles to generate different large numbers of allelic profiles (STs). The extent to which recombination among alleles generates novel STs in *C. trachomatis* is unclear. Although the number of STs per isolate varies, the majority of MLST schemes have been successful for strain discrimination, epidemiologic studies, and evaluation of organism evolution (*10*). MLST, however, may not be sufficiently discriminatory for some epidemiologic investigations, even with increased loci numbers. This may be the case for LGV strains, although our scheme resolved 2 L2b strains from all other LGV strains.

We found that a number of STs for STI isolates were shared across continents. This finding was particularly evident for those from Amsterdam, Ecuador, Lisbon, and San Francisco, which would be expected given increasing opportunities for global travel and international sexual encounters. Notably, L2b isolates (ST33) from proctitis cases differed at 2 loci from other LGV isolates (ST1) and were restricted to Amsterdam. Although some L2b strains from Amsterdam and San Francisco have historically been similar (*30*), ST differentiation most likely reflects the emergence of these strains among men who have sex with men. Not surprisingly, STs for trachoma isolates were restricted to the geographic region of origin where populations travel only locally.

Allele frequencies were assigned on the basis of continental/subcontinental regions (Table 3). Most alleles were observed multiple times, and more than half were region specific. Despite the opportunity for worldwide spread, some strains may be stable within the respective geographic populations. This stability was particularly evident in Africa and Asia, where the frequency of unique alleles was the highest, although this finding also reflects the fact that most isolates were from trachoma populations. As expected, we found, in general, a statistically significant nonuniform distribution of alleles.

Analyses using eBURST and trees constructed in MEGA4 resolved isolates into clonal complexes or clusters. Both methods identified distinctive groupings of strains by disease phenotypes. STIs caused by less common strains formed an eBURST group (CC-B) but were within cluster I on the tree together with the trachoma Subcluster I, which was a separate eBURST group (CC-A). A similar clustering pattern to our tree was found by Pannyhoek et al. (*14*) by using 16 reference and 5 clinical E strains, but they did not distinguish trachoma reference strain B/TW-5 from the LGV group. Our cluster II included only LGV strains. Cluster III contained the noninvasive globally prevalent D/Da, E, and F strains (eBURST CC-C). This cluster represents efficiently transmitted strains with adaptive fitness in the genital tract.

A number of isolates representing different *ompA* genotypes shared the same ST, whereas many isolates of the same *ompA* genotype had different STs (Technical Appendix). Furthermore, 9 isolates were found outside the expected cluster, suggesting that recombinational replacement at the *ompA* locus occurs relatively frequently. Accumulating evidence supports frequent recombination among *Chlamydiaceae*. Initial evidence came from observations of recombination within *ompA* (*4*,*31*) followed by phylogenetic analyses (*32*), and bioinformatic and statistical analyses for multiple species of the family Chlamydiaceae and *C. trachomatis* strains (*15*). Recently, we showed intergenic recombination involving *ompA* and *pmpC*, *pmpE-I*, and frequent recombination throughout the genome with significant hotspots for recombination for recent clinical isolates (*16*,*17*). Pannekoek et al. noted incongruence between *ompA* and *fumC* sequences (*14*). Most recombination in our study involved *yhbG,* *glyA*, and *pykF* (Technical Appendix) with incongruence, compared with *ompA.* Based on *C. trachomatis* recombinants that have been created in vitro, the estimated size of transferred DNA ranged from 123 kb to 790 kb (*33*). Although additional recombination sites may exist in regions that were not sequenced, any gene in our study could be involved in lateral gene exchange with a range of 1,191 bp for a single gene (e.g., *ompA*), 27 kb *(yhbG* to *ompA*) to at least 248 kb (*glyA* to *yhbG*), which is consistent with DeMars and Weinfurter (*33*) and our previous findings (*16*,*17*).

Analysis of the 61 SNPs among the 7 loci showed a statistically significant association of specific polymorphisms with each disease cluster (Table 4). A total of 15 SNPs singly or together identified the LGV cluster. Similarly, 5 SNPs identified the prevalent cluster II D/Da, E, and F strains. Three clinical D and E strains did not contain these SNPs and each appeared to be a recombinant with other STI strains. Only 1 SNP (in *pykF)* identified all trachoma strains in Subcluster I. Reference trachoma strains A and Ba did not contain this SNP, suggesting that they may not represent circulating strains among present-day populations.

Other studies have associated SNPs or indels in *pmp* and *porB* genes with specific disease causing *C. trachomatis* clades (*16*,*34**,**35*). However, SNPs were not individually analyzed for specific disease associations and the target genes encode surface exposed proteins likely to be under selection for epitope variation to avoid immune system surveillance. A frame-shift mutation in 1 of the tryptophan synthase genes, *trpA*, was associated with trachoma strains when compared with all others, although some B and C strains lack the entire gene (*35*). Large deletions in the cytotoxin loci have also been identified that differentiate the 3 disease groups, yet strain B is missing these loci (*36*). The latter study relied on reference strains, which may limit the use of these deletions for identifying disease-specific groups because clinical isolates may vary in deletion size or location. Additionally, tryptophan synthase genes and cytotoxin loci are located within the 50-kb plasticity zone of the chromosome, a region known for genetic reshuffling (*20*). The current study differs from those previously mentioned in that it used housekeeping genes that are not under immune selection or in the plasticity zone. Therefore, the SNPs we identified are probably neutral and can be used as reliable markers for disease association. Furthermore, SNPs were based on reference and clinical isolates of multiples of the same strains from 6 geographic regions, representing a broad diversity of this species.

Given the high rates of infection among STI (*37*) and trachoma populations (*38*,*39*), the ability to distinguish LGV and noninvasive urogenital and trachoma strains, including mixed infections, would aid epidemiologists, clinicians, and public healthcare workers worldwide in determining appropriate therapeutic or intervention strategies (*40*). Our multilocus and SNP typing can now be used to standardize the way an organism is typed; isolates from diverse geographic regions worldwide can be identified and compared; and diverse and emerging *C. trachomatis* strains can be detected for epidemiologic and evolutionary studies among trachoma and STI populations worldwide.

## Supplementary Material

Technical Appendix Predicting Phenotype and Emerging Strains among Chlamydia trachomatis Infections
