# Supplementary material for: Predicting Phenotype and Emerging Strains among Chlamydia trachomatis Infections
Source: Emerg Infect Dis. 2009 Sep;15(9):1385–94. doi: 10.3201/eid1509.090272 (PMC2819883; doi:10.3201/eid1509.090272)
Supplement: Technical Appendix — Predicting Phenotype and Emerging Strains among Chlamydia trachomatis Infections [file 09-0272_Techapp-s1.pdf]

Predicting Phenotype and Emerging Strains among *Chlamydia trachomatis* Infections

Technical Appendix

Technical Appendix Table. Sequence types, allelic profiles, and clinical characteristics of reference and clinical isolates*

		Allele assignment for each locus								
Strain ID†	ST	*glyA*	*mdhC*	*pdhA*	*yhbG*	*pykF*	*lysS*	*leuS*	Region of isolation	Diagnosis
L1/440	1	01	01	03	08	01	04	11	California	LGV
L2/434	1	"	"	"	"	"	"	"	California	LGV
L2/54s	1	"	"	"	"	"	"	"	San Francisco	Proctitis
L2a/UW-396	1	"	"	"	"	"	"	"	Seattle	LGV
L2b/86nl	1	"	"	"	"	"	"	"	Amsterdam	Proctitis
L3/404	1	"	"	"	"	"	"	"	California	LGV
D/84s	2	02	03	03	06	05	04	03	San Francisco	Cervicitis
H/UW-4	3	03	01	03	06	06	04	03	Washington	Cervicitis
A/51t	4	03	03	01	06	03	07	09	Tanzania	Trachoma
H/46nl	5	03	03	02	06	06	08	03	Amsterdam	Cervicitis and vaginal discharge
B/TW-5	6	03	03	03	04	03	05	10	Taiwan	Conjunctivitis
I/UW-12	7	03	03	03	06	01	04	03	Washington	Urethritis
K/UW-31	8	03	03	03	06	02	04	03	Washington	Cervicitis
J/UW36	9	03	03	03	06	02	08	03	Washington	Cervicitis
Ja/UW-92	9	"	"	"	"	"	"	"	Washington	Cervicitis
B/53t	10	03	03	03	06	03	04	09	Tanzania	Trachoma
C/TW-3	11	03	03	03	06	03	05	07	Taiwan	Conjunctivitis
A/48t	12	03	03	03	06	03	05	09	Tanzania	Trachoma
A/59t	12	"	"	"	"	"	"	"	Tanzania	Trachoma
B/50t	12	"	"	"	"	"	"	"	Tanzania	Trachoma
B/60t	12	"	"	"	"	"	"	"	Tanzania	Trachoma
B/61t	12	"	"	"	"	"	"	"	Tanzania	Trachoma
B/62t	12	"	"	"	"	"	"	"	Tanzania	Trachoma
Ba/52t	12	"	"	"	"	"	"	"	Tanzania	Trachoma
C/32n	13	03	03	03	06	03	06	07	Nepal	Trachoma, TS
C/33n	13	"	"	"	"	"	"	"	Nepal	Trachoma, TS
G/15s	14	03	03	03	06	04	04	08	San Francisco	Proctitis
K/42nl	15	03	03	03	06	06	01	06	Amsterdam	Cervicitis w/ vaginal discharge
K/49nl	15	"	"	"	"	"	"	"	Amsterdam	Cervicitis w/ vaginal discharge
J/27s	16	03	03	03	06	06	01	08	San Francisco	Cervicitis/urethritis
E/87e	17	03	03	03	06	06	02	03	Ecuador	Cervicitis
Ba/Apache-2	18	03	03	03	06	06	03	09	Arizona	Conjunctivitis
D/83s	19	03	03	03	06	06	04	03	San Francisco	Cervicitis
H/40nl	19	"	"	"	"	"	"	"	Amsterdam	Cervicitis
H/18s	19	"	"	"	"	"	"	"	San Francisco	Cervicitis/urethritis
I/22p	19	"	"	"	"	"	"	"	Lisbon	Cervicitis/urethritis
J/44nl	19	"	"	"	"	"	"	"	Amsterdam	Cervicitis
D/43nl	20	03	03	03	06	06	04	06	Amsterdam	Cervicitis and vaginal discharge
G/13s	21	03	03	03	06	06	04	08	San Francisco	Proctitis
G/14s	21	"	"	"	"	"	"	"	San Francisco	Proctitis
A/SA-1	22	03	03	03	06	06	05	02	Saudi Arabia	Conjunctivitis
Ia/UW-202	23	03	03	03	06	06	08	03	Washington	Cervicitis
D/2s	23	"	"	"	"	"	"	"	San Francisco	Cervicitis/urethritis
Ia/57e	23	"	"	"	"	"	"	"	Ecuador	Cervicitis
Ia/24s	24	03	03	03	06	06	08	08	San Francisco	Cervicitis/urethritis
Ia/25s	24	"	"	"	"	"	"	"	San Francisco	Cervicitis/urethritis
D/B120	25	03	03	03	06	07	04	01	Washington	Cervicitis
G/16p	26	03	03	03	07	06	08	05	Lisbon	Cervicitis/Urethritis
G/17p	27	03	03	04	06	06	04	03	Lisbon	Cervicitis/urethritis

		Allele assignment for each locus								
Strain ID†	ST	*glyA*	*mdhC*	*pdhA*	*yhbG*	*pykF*	*lysS*	*leuS*	Region of isolation	Diagnosis
Ia/23p	28	03	03	04	06	06	04	04	Lisbon	Cervicitis/urethritis
H/21p	29	03	03	04	06	06	08	03	Lisbon	Cervicitis
G/UW-57	30	03	03	05	06	06	04	03	Washington	Cervicitis
H/20p	31	03	03	07	06	06	08	03	Lisbon	Cervicitis/urethritis
F/38nl	32	04	04	03	02	07	04	03	Amsterdam	Cervicitis and vaginal discharge
L2b/48nl	33	05	02	03	08	01	04	11	Amsterdam	Proctitis
L2b/85nl	33	“	“	“	“	“	“	“	Amsterdam	Proctitis
F/IC-Cal3	34	06	03	03	02	07	04	03	California	Cervicitis
F/8p	34	“	“	“	“	“	“	“	Lisbon	Cervicitis/urethritis
F/9p	34	“	“	“	“	“	“	“	Lisbon	Cervicitis/urethritis
E/19e	34	“	“	“	“	“	“	“	Ecuador	Cervicitis
E/5s	34	“	“	“	“	“	“	“	San Francisco	Cervicitis/urethritis
Ja/41nl	34	“	“	“	“	“	“	“	Amsterdam	Cervicitis and vaginal discharge
Ja/47nl	34	“	“	“	“	“	“	“	Amsterdam	Cervicitis and vaginal discharge
F/10s	35	06	03	03	02	07	04	08	San Francisco	PID
F/11s	35	“	“	“	“	“	“	“	San Francisco	PID
F/12s	35	“	“	“	“	“	“	“	San Francisco	PID
E/39nl	36	06	03	03	03	07	04	03	Amsterdam	Cervicitis
Da/TW448	37	06	03	03	05	07	04	02	Taiwan	Trachoma
D/3s	38	06	03	06	02	07	04	03	San Francisco	Cervicitis/urethritis
E/Bour	39	06	04	03	02	07	04	03	California	Cervicitis
E/45nl	39	“	“	“	“	“	“	“	Amsterdam	Cervicitis
E/28e	39	“	“	“	“	“	“	“	Ecuador	Cervicitis
E/55e	39	“	“	“	“	“	“	“	Ecuador	Cervicitis
E/56e	39	“	“	“	“	“	“	“	Ecuador	Cervicitis
E/6p	39	“	“	“	“	“	“	“	Lisbon	Cervicitis
E/7p	39	“	“	“	“	“	“	“	Lisbon	Cervicitis
E/58t	39	“	“	“	“	“	“	“	Tanzania	Conjunctivitis
E/4s	40	06	04	03	03	07	04	03	San Francisco	Cervicitis/urethritis
Ja/26s	41	06	04	03	05	06	04	08	San Francisco	Cervicitis/urethritis
C/31n	42	07	03	03	01	03	05	07	Nepal	Trachoma, TI
C/35n	43	07	03	03	06	03	05	07	Nepal	Trachoma, TI
C/1n	44	07	03	03	06	03	06	07	Nepal	Trachoma, TI
C/29n	44	“	“	“	“	“	“	“	Nepal	Trachoma, TI
C/30n	44	“	“	“	“	“	“	“	Nepal	Trachoma, TI
C/34n	44	“	“	“	“	“	“	“	Nepal	Trachoma, TI
C/36n	44	“	“	“	“	“	“	“	Nepal	Trachoma, TI
C/37n	44	“	“	“	“	“	“	“	Nepal	Trachoma, TI

*LGV, lymphogranuloma venereum; TS, trachomatous scarring; PID, pelvic inflammatory disease; TI, trachomatous inflammation severe; ST, sequence type.

†Strain ID, first letter refers to the *ompA* genotype; the number after the dash represents the ID# of the clinical strain; the small case letter after the number denotes the geographic region from which the sample was obtained: e, Ecuador; nl, the Netherlands; n, Nepal; p, Portugal; s, San Francisco..

# eBURST report for 87 isolates by group*

Group 1: No. isolates = 29 | No. STs = 19 | Predicted founder = 19

						Average	ST bootstrap	
ST	FREQ	SLV	DLV	TLV	SAT	distance	Group	Subgroup
ST19	5	9	9	0	0	1.5	87%	98%
ST23	3	7	9	2	0	1.72	41%	87%
ST21	2	5	9	4	0	1.94	14%	41%
ST27	1	4	9	5	0	2.05	4%	19%
ST29	1	4	6	7	1	2.27	3%	10%
ST20	1	3	11	4	0	2.05	0%	0%
ST24	2	3	9	6	0	2.16	0%	3%
ST8	1	3	8	7	0	2.22	2%	0%
ST5	1	3	6	8	1	2.38	0%	0%
ST31	1	3	6	8	1	2.38	0%	0%
ST16	1	3	5	10	0	2.38	0%	3%
ST17	1	2	14	2	0	2.0	0%	0%
ST30	1	2	11	5	0	2.16	0%	0%
ST7	1	2	9	7	0	2.27	0%	0%
ST9	2	2	7	8	1	2.44	0%	0%
ST15	2	2	5	11	0	2.5	0%	0%
ST3	1	1	8	9	0	2.44	0%	0%
ST14	1	1	6	8	3	2.72	0%	0%
ST28	1	1	5	11	1	2.66	0%	0%

*ST, sequence type; FREQ, frequency; *SLV, single locus variant; DLV, double locus variant; TLV, triple locus variant; SAT, satellite

Group 2: No. isolates = 19 | No. STs = 7 Predicted founder = 11

						Average	ST Bootstrap	
ST	FREQ	SLV	DLV	TLV	SAT	distance	Group	Subgroup
ST11	1	3	3	0	0	1.5	46%	17%
ST43	1	3	2	1	0	1.66	37%	21%
ST13	2	2	3	1	0	1.83	11%	0%
ST12	7	2	2	2	0	2.0	9%	0%
ST44	6	2	2	2	0	2.0	9%	0%
ST42	1	1	2	2	1	2.5	0%	0%
ST10	1	1	2	2	1	2.5	0%	0%

Group 3: No. isolates = 22 | No. STs = 7 Predicted founder = 34

						Average	ST Bootstrap	
ST	FREQ	SLV	DLV	TLV	SAT	distance	Group	Subgroup
ST34	7	4	2	0	0	1.33	71%	55%
ST39	8	3	3	0	0	1.5	34%	19%
ST36	1	2	3	1	0	1.83	3%	0%
ST40	1	2	2	2	0	2.0	8%	0%
ST35	3	1	3	2	0	2.16	0%	0%
ST38	1	1	3	2	0	2.16	0%	0%
ST32	1	1	2	3	0	2.33	0%	0%

Singletons: size 11
ST26
ST25
ST22
ST18
ST6
ST4
ST2
ST1
ST41
ST37
ST33

*No. of STs, 44; No. of resamplings for bootstrapping 1,000; No. of groups, 3.
